# High resolution simulation of basilar artery infarct and flow within the circle of Willis

**DOI:** 10.1038/s41598-023-48776-0

**Published:** 2023-12-08

**Authors:** Jon W. S. McCullough, Peter V. Coveney

**Affiliations:** 1 Centre for Computational Science, Department of Chemistry, University College London, London, UK; 2 Centre for Advanced Research Computing, University College London, London, UK; 3 Informatics Institute, University of Amsterdam, Amsterdam, The Netherlands

**Keywords:** Fluid dynamics, Cardiovascular diseases, Biomedical engineering

## Abstract

On a global scale, cerebro- and cardiovascular diseases have long been one of the leading causes of death and disability and their prevalence appears to be increasing in recent times. Understanding potential biomarkers and risk factors will help to identify individuals potentially at risk of suffering an ischemic stroke. However, the widely variable construction of the cerebral vasculature makes it difficult to provide a specific assessment without the knowledge of a patient’s physiology. In this paper we use the 3D blood flow simulator HemeLB to study flow within three common structural variations of the circle of Willis during and in the moments after a blockage of the basilar artery. This tool, based on the lattice Boltzmann method, allows the 3D flow entering the basilar artery to be finely controlled to replicate the cessation of blood feeding this particular vessel—we demonstrate this with several examples including a sudden halt to flow and a gradual loss of flow over three heartbeat cycles. In this work we start with an individualised 3D representation of a full circle of Willis and then construct two further domains by removing the left or right posterior communicating arteries from this geometry. Our results indicate how, and how quickly, the circle of Willis is able to redistribute flow following such a stroke. Due to the choice of infarct, the greatest reduction in flow was observed in the posterior cerebral arteries where flow was reduced by up to 70% in some cases. The high resolution domains used in this study permit the velocity magnitude and wall shear stress to be analysed at key points during and following the stroke. The model we present here indicates how personalised vessels are required to provide the best insight into stroke risk for a given individual.

## Introduction

Throughout the world, defects in blood vessels leading to ischemic stroke are a leading cause of death. Indeed, cardiovascular disease has been increasing globally at a steady rate over the last 25 years^1^. These facts make the general study of the circulatory system an important field of ongoing research to better understand physiological factors that may contribute to these statistics.

As medical imaging technology has advanced, it has become possible to obtain images of the vessels throughout the brain in high resolution. This data is also allowing the personalization of numerical simulations of blood flow in cerebral vasculatures to unprecedented levels. Such studies may occur in simulations at all scales of physical realism. For example Benemerito et al^2^ use a 1D model to develop a set of training data for a machine learning model to identify biomarkers for ischemic stroke. Similarly, Padmos et al^3,4^ make use of a 1D model connected to perfusion territories to examine how an acute ischemic stroke impacts flow into the brain tissue beyond the circle of Willis. 3D studies of cerebral blood flow often focus on aneurysms^5,6^ but models for ischemic stroke^7,8^ and general cerebral flow^9^ are also considered. However, the individual variations in the anatomical structure of blood vessels may also be a reason for the relatively generic nature of the risk factors for vascular disease. When an individual’s vasculature may not be imaged until after an ischemic event has occurred, it remains challenging to quantify its risk in advance.

The circle of Willis is an arterial vascular structure located within the brain. Ideally, this structure is fed by the basilar and the left and right internal carotid arteries and is built from connecting communicating arteries that then feed the posterior, middle and anterior cerebral arteries. These outlet vessels then transport blood to the brain tissue beyond. The multiple connections between the vessels of the circle of Willis, in principle, allow the provision of blood to brain tissue to be maintained in the case of a blockage to the vessel. However, Hindenes et al.^10^ observed that the full circle of Willis structure is actually relatively uncommon with it being observed in fewer than one-in-eight cases. Many cases had the circle of Willis separated into two or more components, meaning that redistribution would be impossible if particular vessels were blocked. In support of this, Nouh et al.^11^ note that some 48-58% of the population may have an incomplete circle of Willis. This is a further illustration of why individualised studies may be able to yield better assessments of a given patient’s risk profile to vascular disease.

Within this paper, we investigate how a stroke due to an infarct of the basilar artery presents within an individualised circle of Willis domain. In particular, we are focussed on understanding the transient behaviours immediately following the occurrence of stroke rather than the long-term effects. We will also account for the diversity of circle of Willis structures by repeating our analysis for three physiological variants. We believe that such work will further help to motivate the development of computational techniques in support of developing digital twins of the human body for personalised healthcare.

In Section “Methods” we introduce the HemeLB code that has been used in this study to simulate blood flow in the circle of Willis and describe the cases that we have simulated. Section “Results” presents the results from these studies and they are discussed in Section “Discussion”. The findings of our study are summarised in Section “Conclusion”.

## Methods

To simulate blood flow in this study, we have used the open-source solver HemeLB^6,9,12–16^. The development and optimisation of this code allows it to efficiently solve the 3D macroscopic fluid dynamics in the sparse geometries that are typical of vascular domains. HemeLB exploits the lattice Boltzmann method (LBM) to achieve this in a manner that can be scaled to over 300,000 CPU cores^16^. Unlike finite difference or finite volume techniques that directly solve the Navier-Stokes equations for fluid flow, the LBM uses a discretised form of the Boltzmann equation to replicate these under certain parametrisation and flow conditions, namely in the incompressible limit. The key requirement is that the Mach number of the flow is sufficiently low that error terms can be ignored. In the LBM, macroscopic quantities are solved by taking moments of mesoscopic distributions at each local grid point in the domain. Discussion of the derivation of the LBM and mathematical analysis of the algorithm can be readily found in the wider literature^17–21^. A collision kernel updates the value of these distributions at each time step and a streaming process distributes the updated populations to a selected set of a grid point’s nearest neighbours. The choice of this set influences both the accuracy of the implementation and the memory demands of computations. This ability to have updates made locally makes the LBM relatively straightforward to parallelise for execution on multicore computing architectures. With the distributions being the fundamental variables stored at each lattice site, boundary conditions for the implementation of velocity and pressure need to be constructed in terms of these quantities. In HemeLB, several boundary conditions are available but we utilise the methods described by Nash et al.^15^ to achieve this in the current study. In this paper, Nash et al. also present studies on the convergence behaviour of HemeLB against analytical results. The HemeLB code has been validated in a number of previous works examining vascular flow^9,22,23^. We particularly refer to the work of Groen et al.^9^ to illustrate how HemeLB can accurately capture flow behaviour in complex vascular domains. We utilise these results to illustrate the validity of the HemeLB blood flow solver for cerebral vascular flows and build upon this established body of work within the current paper.

**Figure 1:**
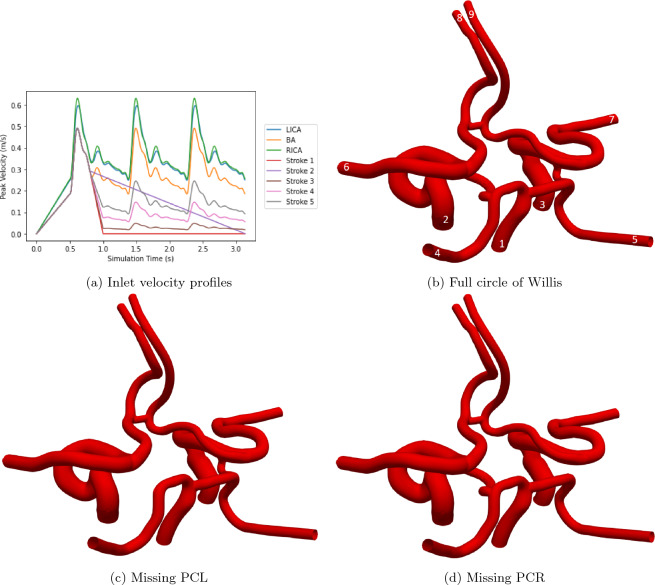
(**a**) Inlet profiles provided to the healthy basilar artery (BA), left and right internal carotid arteries (LICA, RICA). A stroke in the basilar artery has been simulated through five different inlet profiles being applied to this inlet (Stroke 1–5). (**b**)–(**d**) Circle of Willis geometries examined in this study. In (**b**), the numbering of arteries is as follows: 1 = basilar artery, 2 (3) = left (right) internal carotid artery, 4 (5) = left (right) posterior cerebral artery (PCA), 6 (7) = left (right) middle cerebral artery (MCA) and 8 (9) = left (right) anterior cerebral artery (ACA).

In this paper, we study flow through the circle of Willis by driving inlet flow to the basilar and left and right internal carotid arteries with prescribed velocity profiles. With Nouh et al.^11^ observing that some 20% of ischemic strokes involve the posterior circulation of the circle of Willis, a stroke has been simulated by modifying the inlet profile to the basilar artery. We have generated five different infarct scenarios and have compared the changes in flow through the circle of Willis to that generated by a healthy flow profile. These are illustrated in Fig. 1 and have been chosen to represent a sudden stop to flow, a slow stoppage to flow and three levels of restriction to the flow in the basilar artery. In the final three cases, the flow is restricted to 50%, 30% and 10% of the original flow. All reductions to flow are imposed in a linear fashion from the same moment in the inlet profile. We have chosen this approach as, although it may present some minor instantaneous deviations from the presentation of a physical stroke, it is able to simply approximate the overall behaviour over several heartbeats. All applied inlet profiles are transient in nature and are based on the velocity traces applied in Patronis et al.^22^. After a small warm-up period to assist in numerically initialising flow within the domain the applied profiles last for three heartbeats of physical time (approximately 2.6s). At each inlet plane, the local velocity at each lattice site is determined by applying a weighting factor to scale the peak velocity. These factors have been calculated through a parabolic approximation based on the distance between the local site and the vessel wall. The site furtherest from the wall has a weight of one and sites at the wall a weight of zero. In a circular domain, this strategy reconstructs a Poiseuille flow profile.

Outlet flow at the left and right anterior cerebral arteries, left and right middle cerebral arteries, and left and right posterior cerebral arteries were controlled by fixed pressure boundaries using the approach of Nash et al.^15^ that implicitly enforces a pressure gradient of zero at the outlet planes. This approach has been chosen following previous studies with HemeLB that successfully capture cerebral blood flow^9,22^. The choice of boundary conditions can potentially have significant impacts on the observed flow within a simulation. Windkessel-type boundary conditions are commonly used for vascular studies (particularly for 1D models and for finite volume methods) but are less widely applied to LBM simulations. In this study we chose the simpler (and proven) boundary condition to implement and tune. With the use of the LBM in these study, we are also more interested in examining the dynamic characteristics of the blood flow.

As the circle of Willis structure is highly variable between individuals, we have modified the full domain provided by Figueroa^24^ so as to also have the left and right posterior communicating arteries (PCL and PCR respectively) removed. These 3D domains were generated from Computed Tomography Angiography (CTA) scans of an individual patient’s vessels^24^. As the modified vessels were derived from the full domain, they do not represent a particular individual but still represent the nature of personalised vessels for simulation purposes. These domains measure approximately 7$\times$5.5$\times$3.9 cm. According to the survey conducted by Hindenes et al.^10^ these three arrangements represent 31.4% of the participant group. It should be noted that in this study the complete circle of Willis was only the third most common variant of the 47 identified, as it was seen in just 11.9% of participants.

The simulations described here were conducted on SuperMUC-NG located at the Leibniz Supercomputing Centre, Germany. The circle of Willis domains were discretised with a lattice spacing of $\delta x$ = 25 $\upmu$m, leading to domains of approximately 170 million lattice sites. A grid independence study with the full circle of Willis domain discretised to $\delta x$ = 15 $\upmu$m was conducted with only marginal differences to the coarser domain, justifying our use of the lower resolution case. A time step of $\delta t$ = 1 $\upmu$s was used with a single relaxation time LBM collision model. This led to a relaxation time of 0.5192. Jobs were run for a total of 3 million iterations for a simulation of 3 s of physical time. When run on 7200 cores (150 nodes) of SuperMUC-NG, individual jobs completed in around 10 h.

## Results

**Figure 2:**
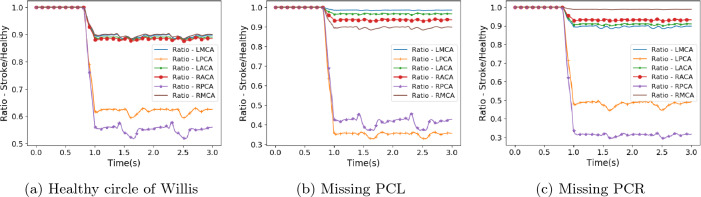
Flow rate at circle of Willis outlets compared to that observed in healthy flow for stroke scenario 1.

**Figure 3:**
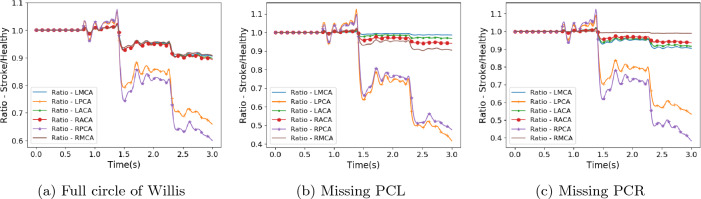
Flow rate at circle of Willis outlets compared to that observed in healthy flow for stroke scenario 2.

**Figure 4:**
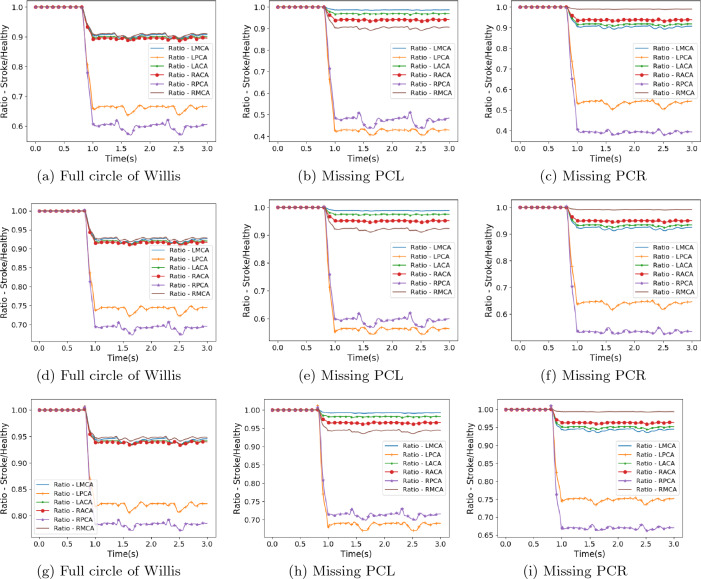
Flow rate at circle of Willis outlets compared to that observed in healthy flow for stroke scenarios 3 (a–c), 4 (d–f) and 5 (g–i).

The harm done by cerebral stroke is due to the reduction of blood flow to regions beyond the infarct. In this section, we present the key results obtained and derived from our numerical simulations of cerebral stroke with HemeLB. We provide some commentary on how these were generated and postpone the discussion of the consequences of these results to the Discussion. In Figs. 2, 3 and 4 we compare the flow simulated to be leaving the circle of Willis in our selected stroke scenarios with that observed with a healthy flow of blood entering the domain.

**Figure 5:**
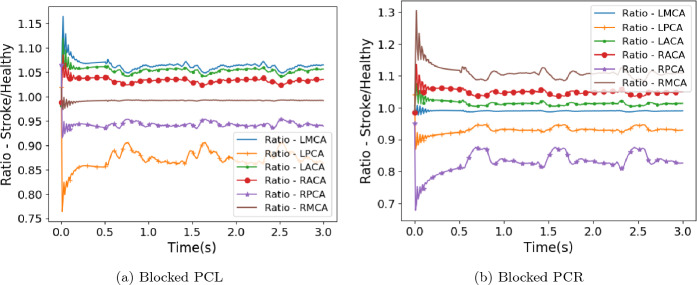
Flow rate at circle of Willis outlets compared to that observed in healthy flow by regarding the models of missing posterior communicating arteries as blocked vessels.

As a corollary to the cases of basilar stroke presented above, comparing the normal flow rates in the full circle of Willis and those in the modified geometries also provides insights into the case of stroke due to a blockage of the posterior communicating arteries. In both cases we use the results generated with the healthy profiles for analysis. Functionally, the absent vessels in the modified geometries represent a complete blockage of the relevant posterior communicating artery. With the modified geometries having smooth walls where the vessels originally connected, this represents the blocked vessel being filled with stagnant fluid. The outlet flow results of this comparison are presented in Fig. 5.

**Figure 6:**
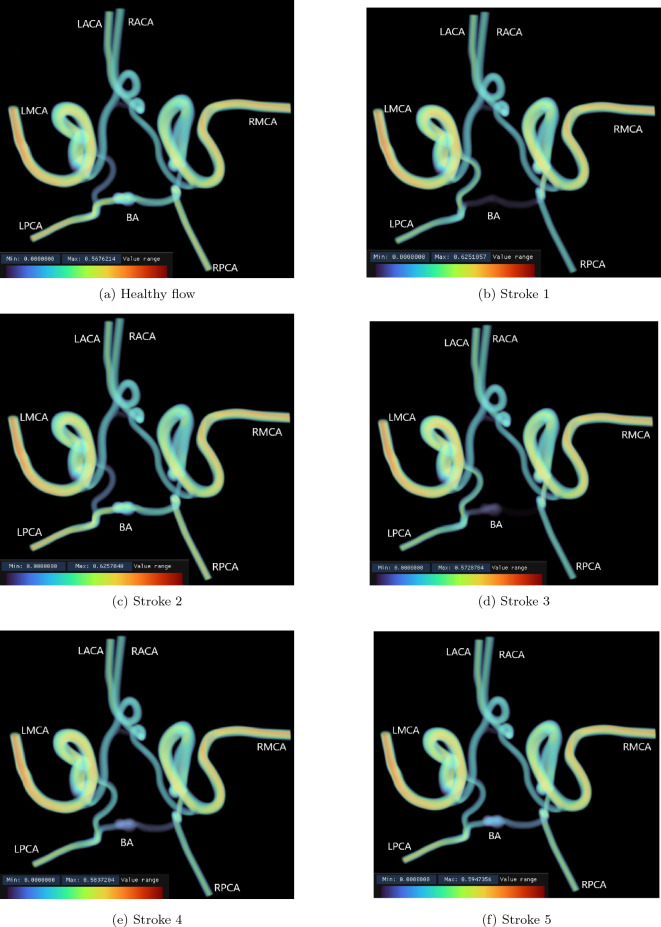
Velocity magnitude distribution within the full circle of Willis after one physical second of simulation time. The maximum value of each flow scenario at this point varies between approximately 0.595 ± 0.03 m/s. These plots illustrate the change in flow through the vessels of the circle of Willis following a stroke. In particular, the activation of the connecting vessels between the MCA and PCA vessels following a stroke compared to the healthy case is clearly observed.

The use of high resolution 3D simulations also allows for detailed images of the stroke occurring to be generated. In Fig. 6 we capture the 3D distribution of velocity magnitude within the full circle of Willis domain for each of the input flow profiles being considered. These were created using the Intel OSPRay tools developed for processing HemeLB output data on SuperMUC-NG^25^.

**Figure 7:**
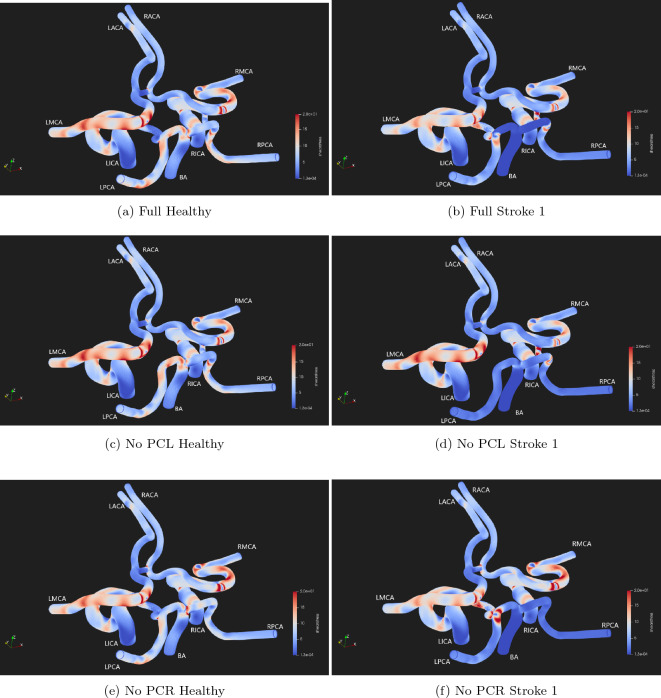
Shear stress magnitude measured at the wall surfaces of the test domains under the test conditions of Healthy and Stroke 1 flow measured after approximately 1.5 s of simulation time. Values between 0.0 and 20.0 Pa are presented to allow contrasts to be identified.

**Figure 8:**
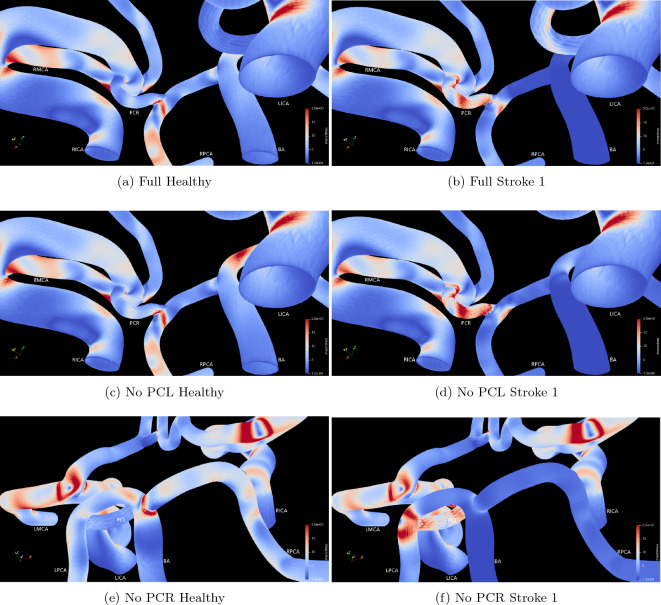
Shear stress measured at the wall surfaces of the test domains under the test conditions of Healthy and Stroke 1 flow measured after approximately 1.5 s of simulation time. (**a**–**d**) focus on the PCR connecting vessel between the RICA and RPCA whilst (**e**–**f**) focus on the basilar artery bifurcation. Values between 0.0 and 20.0 Pa are presented to allow contrasts to be identified.

The use of such simulation domains also allows for the shear stress across the vessel walls to be observed. In Figs. 7 and 8 we present the shear stress observed in each of the three domains after 1.5 s of simulation time. This point roughly coincides with the maximum velocity of the second full heartbeat of the healthy flow profiles.

## Discussion

Due to the choice of blocking the basilar artery, the geometry of the circle of Willis dictates that the reduction in flow will be greatest in the left and right PCAs and this has been observed throughout the stroke scenarios studied. Stroke scenario 1 can be regarded as the most severe of the variants examined with blood flow completely halted within around quarter of a heartbeat. When the full circle of Willis is present, the flow in the RPCA is reduced to around 55% of the healthy value and the LPCA is reduced to around 62%. The remaining cerebral arteries are reduced to around 90% of their respective values. When the same stroke pattern is applied to the circle of Willis structures with missing posterior communicating arteries, the distribution of flow changes significantly. Firstly, the flow in the PCA on the same side as the missing communicating artery is reduced to 30–35% of the healthy flow. The flow in the alternate PCA is reduced to between 40 and 50% of the healthy flow. This reduction is greater for the missing left communicating artery, possibly due to the right posterior communicating artery being connected less favourably to the RICA in the geometries studied. The flow in the middle and anterior cerebral arteries is observed to be greater when the posterior communicating artery is missing than when the full circle of Willis is present. This is a result of the PCAs needing to be supplied with blood by only one of the communicating arteries. The relative sizes of the communicating arteries may also limit how much the more distant feeding arteries are able to supplement flow to the PCAs.

The characteristic trends observed in the stroke 1 scenario are repeated in the remaining four stroke scenarios where the blood flow coming from the basilar artery either ramps down to zero flow or is merely reduced to a proportion of the healthy flow value. With some flow volume still being provided by the BA, the reductions in flow rate to the PCAs is not as severe. The flow to the MCAs and ACAs remains broadly at the same range of values despite the changes in inlet flow profiles.

The outlet flow rate behaviour of the stroke 2 scenario (Fig. 3) is qualitatively quite different to that seen in the other patterns. As can be seen in Fig. 1a, the stroke 2 profile is enforced as a linear reduction from the point of stroke to the end of the simulation. During the second half of the first heartbeat (approximately 0.9–1.5 s of simulation time) this yields some instances where the flow rate of the stroke profile is greater than that of the healthy flow and the consequence of this is reflected in the outlet ratios being greater than unity during this period. This is not seen in any of the other stroke scenarios. The oscillations of the observed ratios throughout the simulations are similarly more pronounced for this profile due to the relative flow rates between the healthy and stroke profiles transiently changing rather than being steady as is the case in the other stroke scenarios.

In the corollary cases, where a missing posterior communicating artery is interpreted as a complete blockage to this vessel, the reduction in flow to the PCAs is far less severe than compared to the cases described above. Here the flow remains above 80% of the original value for the PCA on the same side as the blockage and above 90% for the opposite vessel. In the absence of the communicating arteries, the flow leaving the ACAs and MCA on the same side as the blockage actually increases by between 5 and 15%. Flow through the MCA on the opposing side to the blockage remains basically identical to that observed in the full circle of Willis. This case is slightly different to our other studies in that we are not triggering a stroke, rather we are examining a more stable long term state of no flow through the blocked communicating artery. In reality, there may be some movement of blood within the vessel either side of the blockage (unlike the completely stagnant flow assumed in our construction) but we expect the changes to overall outlet flow due to this to be small.

The flow velocity images (Fig. 6) indicate the role that the communicating arteries play in supplying flow to the PCAs in the case of a blockage to the BA in the full circle of Willis domain. In particular, the activation of the connecting vessels between the MCA and PCA vessels following a stroke compared to the healthy case is clearly observed. As seen in Figs. 2, 3 and 4, the reduction in flow is greatest in the RPCA and these flow renderings highlight how the bias in flow from the BA towards the LPCA serves to exacerbate this. Stroke 3 particularly illustrates this with the entirety of the flow from the BA travelling to the LPCA. It can also be observed that there is no change in flow crossing between the left and right sides of this circle of Willis as a result of the blockage of the BA as can be observed through the lack of change in flow speed in the connecting vessel between the LACA and RACA. The Hindenes et al.^10^ study observed that approximately 2.6% of the observed population were missing this vessel and these results suggest that such patients may have similar susceptibility to BA stroke as those with a full circle of Willis. These images also illustrate how relatively minor the changes in flow are to the rest of the domain. The maximum value of each flow scenario at this point remains fairly consistent between each flow situation, being seen to vary between approximately 0.595 ± 0.03 m/s. However, flow is seen to be accelerated in all stroke cases compared to the healthy scenario.

The images of wall shear stress (Figs. 7 and 8) also clearly indicate how a stroke in the BA manifests as flow changes throughout the circle of Willis. Several changes in the shear stress distribution are clearly observable at the time of the peak input velocity (Fig. 7). The peaks of shear stress throughout the outlet vessels have all been reduced as a consequence of the overall reduction in flow caused by the blockage of the BA. The most significant observable increase in shear stress occurs within the posterior communicating vessels and especially where these join with the major vessels. The absence of flow in the basilar artery has reduced virtually all of the shear stress within this vessel and up to the junction with the posterior communicating arteries. In Fig. 8, we highlight some specific regions where the change in shear stress magnitude between the vascular morphologies and flow pattern are particularly notable. The increase in flow resulting from the redirection of flow through the PCR connecting vessel as a result of the blockage of the basilar artery manifests in a significant increase in the shear stress observed in this vessel—seen through the comparison of Fig. 8a–d. Comparison of Fig. 8a,c also reveal that in healthy flow, the absence of the PCL vessel does not result in significant differences in shear stress within the PCR vessel. However, once the basilar artery is blocked the intensity of shear stress throughout the PCL is significantly increased as a result of the absent PCR (Fig. 8b,d). In Fig. 8e,f we focus on the changes in shear stress magnitude around the bifurcation of the basilar artery in the absence of the PCR vessel. Between these cases, stress in the right-side vessels remains largely unchanged. However in the left hand vessels, the diversion of flow through the PCL reduces the intensity of shear stress towards the LMCA and causes a significant increase in shear stress in the PCL and, particularly, on the wall opposing its junction with the LPCA. Over the long term, this change in shear stress distribution may lead to further complications in addition to the loss of blood flow feeding the brain due to the original stroke.

Indeed, the influence of wall shear stress, and changes in its distribution, are implicated in a number of vascular diseases^26^. As noted by Holland et al.^26^, wall shear stress has been identified as a factor in the potential initiation and rupture of aneurysms, and in the build-up of plaque deposits within vessels. In our study, we have considered the vessels to remain at a constant diameter throughout the healthy and stroke waveforms. Changes in wall shear stress activate cellular signalling pathways that control the dilation or constriction of blood vessels to help maintain flow within normal bounds^26,27^. It has been observed that this process can take on the order of seconds to minutes to lead to the maximal vessel changes^28,29^. Given that our simulations only extend less than three seconds beyond the onset of stroke, we believe that this mechanism would have had minimal impact on the outcomes presented in our studies. This said, it is an extremely important consideration that would need to be estimated and tracked for vascular simulations of longer duration post-stroke to ensure that the correct vascular geometry is being assessed.

In Hindenes et al.^10^, 47 variations of circle of Willis structure are identified in a survey of over 1800 participants. Of these, 34 had the circle of Willis split into two or more distinct segments, representing over 60% of the participants. Individuals with such vascular morphology may be considered to be at greater risk of harm from certain types of stroke as flow is not able to be effectively redistributed in the case of a blockage. In many cases, blockage of feeder arteries would entirely prevent flow to sections of the brain. For example, in the most commonly observed variant (27.8%) in the study, where both posterior communicating arteries are missing, the blockage of the basilar artery as studied in our work would lead to no blood flow feeding the sections of the brain fed by the posterior cerebral arteries. As the circle of Willis structure of a patient may not be known ahead of a stroke occurring, it may be challenging to fully quantify the risk of severe medical outcomes for an individual patient. With many data-driven models being focussed on assessing the risk profiles of patients for stroke and other serious cardiovascular conditions^2^, such anatomical variations among the populations need to be accounted for to reduce the risk of erroneous forecasts.

In this study, we have used the measure of flow leaving the circle of Willis via the six cerebral arteries as a proxy for the amount of blood flow reaching brain tissue. In practice, the network of arteries beyond the circle of Willis modelled here will provide further pathways for the redistribution of blood in the case of a blockage. However, as these pathways may be much smaller than the vessels considered here, the volume of blood that can be transferred by such mechanisms may be limited. Numerical studies have examined the effect of such networks and collaterals on the distribution of blood^3,4^ within a brain during stroke. Quantification of an individual’s vascular connectivity ahead of a stroke can be difficult to obtain and remains largely an unknown factor in the prediction of a patient’s prospects from a stroke.

Our simulation results illustrate that reduction in blood flow to brain tissue is an almost instantaneous consequence of a blockage to a major vessel in the circle of Willis. This serves to reinforce the message that rapid diagnosis and treatment of such blockages is essential to reduce the risk of negative outcomes from a stroke. In a future study, we plan to examine how the removal of the blockage—either instantaneously or gradually—impacts the re-establishment of normal flow through the circle of Willis.

## Conclusion

Cerebro- and cardiovascular disease is a leading cause of disability and disease throughout the world, and has been increasing in prevalence over the last 25 years. In this paper, we have used the 3D blood flow simulation tool HemeLB to study blood flow within three physiological variants of the circle of Willis following an infarct of the basilar artery. The use of an explicit, high-resolution modelling framework allows us to capture the redistribution of flow during, and immediately following, the occurrence of the stroke. This is in contrast to other approaches that may take a more long-term view of stroke impact. Through comparison to a healthy input profile, our work highlights exactly how the flow leading away from the circle of Willis to the cerebral arteries is reduced. We particularly observed that, due to the choice of infarct in the basilar artery, outflow from the posterior cerebral arteries was reduced by the largest amount—up to approximately 70% in some cases. Asymmetries caused by the absence of the left or right posterior communicating arteries mean that flow in the opposite middle cerebral artery remained almost unchanged. As circle of Willis physiology can vary significantly between individuals, these results highlight how a nominally identical stroke can cause very different stroke outcomes. The use of 3D domains allows the generation of detailed maps of velocity magnitude and shear stress throughout the individualised geometries used in this study. This allows particular areas of change to be clearly identified and, if used in a clinical setting, may permit the risks of further vascular pathology to be identified. We hope to extend the work conducted here by both studying the re-establishment of flow following the removal of the infarct and examining the changes caused by the inclusion of a more realistic model for vessel wall boundaries^30^.

## Data Availability

The datasets used and/or analysed during the current study available from the corresponding author on reasonable request.
